# Burnout, Depression, and Job Stress Factors in Healthcare Workers of a Romanian COVID-19 Dedicated Hospital, after Two Pandemic Years

**DOI:** 10.3390/ijerph20054118

**Published:** 2023-02-25

**Authors:** Violeta Briciu, Daniel-Corneliu Leucuta, Gyöngyvér Erika Tőkés, Doina Colcear

**Affiliations:** 1 Department of Infectious Diseases, Iuliu Hatieganu University of Medicine and Pharmacy, 400348 Cluj-Napoca, Romania; 2 The Clinical Hospital of Infectious Diseases, 400348 Cluj-Napoca, Romania; 3 Department of Medical Informatics and Biostatistics, Iuliu Hatieganu University of Medicine and Pharmacy, 400349 Cluj-Napoca, Romania; 4 Department of Applied Social Sciences, Faculty of Technical and Human Sciences from Târgu Mureş, Sapientia Hungarian University of Transylvania, 540485 Targu Mures, Romania

**Keywords:** COVID-19, healthcare workers, burnout, depression, occupational stress

## Abstract

The COVID-19 pandemic put unprecedented pressure on all areas of activity, especially healthcare workers. Understanding the psychological response to the pandemic in healthcare workers is an important challenge. This study aims to investigate burnout, depression, and job stress factors in the medical personnel of a COVID-19-dedicated hospital, two years after the beginning of the pandemic. The survey was performed between the fifth and sixth pandemic waves in Romania. Employees of the Clinical Hospital for Infectious Diseases, Cluj-Napoca, completed an online survey using four tools: Maslach Burnout Inventory (MBI), Copenhagen Burnout Inventory (CBI), the Karasek Job factors questionnaire, and the Patient Health Questionnaire–9 (PHQ-9). A total of 114 employees completed the questionnaire (10.83% of total employees). The results showed 100% prevalence of Maslach burnout (56.1% moderate and severe burnout) and 63.1% prevalence of depression. The infectious disease resident doctors had the highest prevalence of burnout scores, depression, and perceived Karasek job demands. The 22- to 30-year-old age group and the group with fewer than ten years of professional experience had a significantly higher prevalence of burnout and depression than older employees or employees with more professional experience. The COVID-19 pandemic continues to have a high impact on the mental health of healthcare workers.

## 1. Introduction

The COVID-19 pandemic has generated an unprecedented global health crisis. In the face of uncertainty, healthcare workers continue to provide care under physically and emotionally stressful conditions that continue to evolve in parallel with the changing landscape of the pandemic itself. Even in non-pandemic times, healthcare workers are faced with highly stressful work daily, and as the pandemic started, they were exposed to an extreme rise in daily work-related stressors, witnessing severe illness and death at unprecedented rates, and experienced threats to their own safety. Understanding the psychological response to the pandemic in healthcare workers is an important challenge.

Burnout is a real and serious health problem that has been increasingly recognized and documented in the past years. The burnout construct is defined as a psychological syndrome caused by a prolonged response to interpersonal stressors, mainly at the workplace, which encompasses three dimensions: emotional exhaustion, depersonalization, and a decrease in personal achievements [1,2]. Recently, The World Health Organization (WHO) has included burnout in the International Classification of Diseases (ICD-11) in the section ‘Factors influencing health status or contact with health services’ under the definition of ‘Burnout’ (QD85) [3]. In ICD-11, the syndrome of burnout has the same three dimensions defined by Maslach and Leiter in 2016 [2], but the description differs slightly and includes: (1) feelings of energy depletion or exhaustion; (2) increased mental distance from one’s job, or feelings of negativism or cynicism related to one’s job; and (3) a sense of ineffectiveness and lack of accomplishment. Burnout refers specifically to phenomena in the occupational context and should not be applied to describe experiences in other areas of life [3]. This multidimensional paradigm of burnout emphasizes the importance of individually experiencing stress in the workplace and involves the perception of oneself and others [2]. Among the consequences of burnout, a high number of absences from work were reported due to mental disorders and disorders of the circulatory, respiratory, and musculoskeletal systems. Exhaustion was more closely related to circulatory system diseases and cynicism to digestive system diseases [4].

Burnout is present in all professions and all countries. A Swedish study carried out in 2002 on 6118 people established that burnout with symptoms in all three dimensions was generally associated with depression, anxiety, sleep-related disorders, memory impairment, and neck pain or back pain, as opposed to simple exhaustion, in which no such associations were found [5]. In 2015, the European Working Conditions Surveys (EWCS) published ‘The sixth European Working Conditions Survey (EWCS)’, organized by Eurofound. It showed that burnout prevalence was 17% in countries outside of the European Union and 10% in European countries (8.1% in Romania) [6]. The results of the European Working Conditions Telephone Survey 2021, carried out between March and November 2021 with over 70,000 interviews in 36 countries, are close to being published (Eurofound, 2022, forthcoming). This survey collected longitudinal data for monitoring job quality, and the working paper was published in November 2022 [7]. It presented an index of work engagement that ranged from 0 (lowest) to 100 (highest). The survey found that between 2020 and 2021, all respondents’ means dropped from 74.3 to 72.1 for this index, indicating that people became less engaged, mainly during the COVID-19 pandemic. The results regarding burnout have not yet been published.

One of the most well-studied areas of activity from the point of view of burnout is the healthcare system [8,9,10,11,12,13]. In a 2012 study comparing general US workers with US doctors, doctors were more likely to have symptoms of burnout (37.9% vs. 27.8%) and to be dissatisfied with the balance of their professional and working lives (40.2% versus 23.2%) [8]. A 2020 review found that about one in three doctors experience burnout at some point, or 37.9%, compared to 27.8% in the control population [9].

The COVID-19 pandemic generated increased stress and anxiety in healthcare workers, caused by the risk of infection and death due to COVID-19, the risk of infection of loved ones, self-imposed quarantine, social isolation, prolonged work shifts, a lack of specific COVID-19 protocols, lack of protective equipment, reduced holidays, diminishing doctor–patient relationships due to telemedicine practice and worrying about being asked to care for patients in more critical conditions than they are trained for, and limited availability of up-to-date scientific data [10]. Other causes were a lack of quick access to testing, fear of spreading the disease in the workplace, and uncertainty regarding whether the organization will support or care for them or their family’s needs if they become infected [11]. In addition to those mentioned, the long-term unknown effects of COVID-19, the uncertainty that came with each new wave of the pandemic due to new variants of the virus, and the impact of critical staff shortages due to colleagues becoming sick and leaving work have had a strong effect [12].

Due to the psychological impact of the COVID-19 pandemic, WHO published messages to be used to support mental and psychosocial well-being for the general population, healthcare workers, team leaders or managers in health facilities, carers of children, older adults, and people with underlying health conditions [13].

At the beginning of the pandemic, intensive care was the most affected in terms of professional stress due to COVID-19. The first study, conducted between March and April 2020 and involving 9492 people (doctors, resident doctors, nurses, and pharmacists in the field of intensive care), showed that the perceived stress level was 8 (level 10 representing an extreme stress level) compared to the level before the pandemic, which was 3 [14].

The COVID-19 pandemic put a high level of pressure on the healthcare system in Romania. At the end of October 2021, during the wave caused by the Delta variant of concern, Romania held the first position globally in terms of daily new COVID-19 deaths per million persons [15]. There is a previous study that evaluated burnout in Romanian healthcare workers. A study conducted in 2021 in an infectious disease hospital showed that, 12 months after the admission of the first COVID-19 patient, 61.86% of the staff had medium or high levels of burnout [16].

The main objective of the present study was (1) to evaluate the prevalence and severity level of burnout, depression, and occupational stress among the first-line medical staff that managed COVID-19 patients two years after the start of the pandemic in Romania. The secondary objectives were (2) to investigate the presence and degree of association between respondents’ characteristics (profession, age, professional experience, gender, and leadership position) and burnout, depression, and occupational stressors, as well as (3) the multivariate relation between Maslach burnout score and sex, age, and profession.

## 2. Materials and Methods

### 2.1. Study Design

The design of the study was observational. We conducted a cross-sectional survey among employees of the Clinical Hospital for Infectious Diseases, Cluj-Napoca.

### 2.2. Setting

The Clinical Hospital of Infectious Diseases Cluj-Napoca is a tertiary mono-specialty hospital that provides medical services for patients with infectious pathologies from Cluj County and neighboring counties.

Starting from March 2020, the 200-bed hospital (with an ICU unit of 10 beds, extended to 20 beds during the pandemic) was transformed by a Health Ministry order into the first-line hospital for COVID-19 patients in Cluj County, dedicated exclusively to COVID-19. The first COVID-19 patient was hospitalized on 28 February 2020, being the second patient reported in Romania.

The study took place between the 4th of May and the 17th of June 2022, the interval between the fifth and sixth waves of the COVID-19 pandemic in Romania [14]. Before the 4th of May, there were 9403 COVID-19 patients hospitalized in the medical unit and 527 deaths due to COVID-19.

Permission to conduct the survey was granted by the hospital’s ethical committee on the 3rd of May, 2022 (permission number. 8101).

### 2.3. Participants

All hospital employees were invited to participate in the study, all being considered eligible. Participation in the study was voluntary. The recruitment method was simple, with an e-mail invitation after a short presentation of the study’s objectives in an online meeting explaining the purpose and name of the investigators. The participants were told that they could benefit from a psychiatric consultation and free recommendations provided by one of the investigators with expertise in the field. We converted the paper’s questionnaires into online questionnaires via Google Forms. Questionnaires were completed online, all data being centralized by the investigators in an Excel file.

### 2.4. Data Measurement

#### 2.4.1. Validated Psychometric Instruments

In order to reliably quantify burnout, depression, and occupational stress, we identified appropriate scales that were validated: for burnout, Maslach Burnout Inventory (MBI) and Copenhagen Burnout Inventory (CBI); for occupational stress, the Karasek job factors questionnaire; and for depression, the Adult Depression Severity Scale, adapted from the PHQ-9.

The Maslach Burnout Inventory (MBI) is the golden standard for measuring occupational exhaustion that can occur, according to the classical definition, in people who work ‘with people’ where there is a high emotional charge [17]. It was developed in the form of a questionnaire with 25 items that measured three areas of burnout: Maslach emotional exhaustion (9 items), Maslach depersonalization (6 items), and Maslach personal achievement reduction (10 items). A 6-step Likert scale was used as a response, as follows: 0—never, 1—very rare, 2—rare, 3—sometimes, 4—frequent, and 5—very common [1]. Each score of the sub-scale was calculated by combining all the scores of the items with the specification that certain specific domain items are reverse-scored [17,18].

The Copenhagen Burnout Inventory (CBI) was developed as an alternative due to the fact that the Maslach scale includes two items that do not represent psychological manifestations of professional exhaustion, but rather the coping mechanism (depersonalization) and the long-term consequence of fatigue (reduction in personal achievements) [19]. In CBI, the core of burnout is fatigue and exhaustion, as defined by Schaufeli and Greenglass (2001) as ‘a state of physical, emotional, and mental exhaustion resulting from long-term involvement in emotionally demanding work situations’ [20]. CBI contains three dimensions: CBI personal burnout (6 items), CBI work-related burnout (7 items), and CBI client-related burnout (clients, patients, social service recipients, elderly citizens, or inmates) (6 items). For questions 1–6 on the CBI personal burnout scale; for questions 4, 5, 6, and 7 on the CBI work-related burnout scale; and for questions 5 and 6 on the CBI client-related burnout scale, a 5-step Likert scale was used for the responses: 100—always, 75—often, 50—sometimes, 25—rarely, and 0—never/almost never. For questions 1, 2, and 3 on the CBI work-related burnout scale and for questions 1, 2, 3, and 4 on the CBI client-related burnout scale, the following 5-step Likert scale was used: 100—a very high degree, 75—a high degree, 50—somewhat, 25 –a low degree, and 0—a very low degree.

The Karasek job factors questionnaire was adapted according to the Job Demand–Control–Support (JDCS) model and includes the following areas: (a) Karasek job demands (9 items), (b) Karasek job control (9 items), and (c) Karasek social support (8 items). It does not include physical requirements or workplace insecurity, which are included in the Job Content Questionnaire (JCQ) [21]. The Karasek job demands subscale (a) refers to the quantity–rapidity, complexity–intensity of work, and interruptions–predictability of work. The Karasek job control subscale (b) refers to freedom and limits of decision, freedom of action, and development of professional skills. The Karasek social support subscale (c) reflects professional and emotional support from superiors and colleagues. A 4-step Likert scale was used for responses: 1—never true, 2—often not true, 3—often true, and 4—always true.

The Adult Depression Severity Scale (9 items), adopted by the American Psychiatric Association (APA) according to the Patient Health Questionnaire-9 scale (PHQ-9) is a very useful tool to monitor depression [22]. The 9 items are rated on a Likert scale in 4 steps from 0 to 3 (not at all = 0, on some days = 1, half of the days = 2, and almost every day = 3).

The cut-off values for the previously presented scales for identifying score severity levels, are presented in Table 1. 

#### 2.4.2. Demographic and Professional Characteristics

Besides the psychometric instruments, we collected data regarding age, sex, profession, management position, and years of work in the current profession.

### 2.5. Statistical Analysis

The data obtained from the questionnaires were analyzed using IBM SPSS Statistics 23.0 and R environment for statistical computing and graphics (R Foundation for Statistical Computing, Vienna, Austria), version 4.1.2. Variables measured at a nominal scale were represented using proportions (%), and quantitative variables were presented as means (SD). The *t*-test was used on independent samples to compare the scores of the two independent groups. The average scores of each field were compared using the one-way ANOVA test with independent samples, and we measured F statistic, *p*-value, and ƞ^2^ (effect size). The F value in one-way ANOVA is a tool to help you answer the question, ‘Is the variance between the means of two populations significantly different?’ The F value in the ANOVA test also helps to compute the *p* value that indicates whether the null hypothesis should be rejected. The effect size, calculated by dividing the sum of squares between groups/sum of squares total, represents the magnitude of the difference between groups. Post hoc Bonferroni correction was used for multiple comparisons. A multiple linear regression model with the natural logarithm of the Maslach burnout score was used as the dependent variable, and sex, age, and profession were used as independent variables. A natural logarithm was used to correct the normality of residuals as assessed with a quantile–quantile plot of residuals. The years of work variable was not included in the model due to the high correlation and multicollinearity with age, as observed with the variance inflation factor and Pearson correlation coefficient. Since there was a degree of heteroskedasticity, we used robust 95% confidence intervals computed with sandwich estimators, as well as 900 replications of bootstrapping with bias-corrected and accelerated confidence intervals. The functional form of continuous variables with the dependent variable was assessed with component and residual plots. A 5-fold cross-validation of this model, repeated 200 times, was performed to assess its performance. A *p* < 0.05 value was considered statistically significant.

## 3. Results

### 3.1. Characteristics of Participants

Out of 1052 hospital staff, 10.83% (114) of the employees participated in the study. The majority were women (82.50% women vs. 17.50% men). The respondents’ mean age ± standard deviation (SD) was 41.38 ± 10.60 years, ranging from 22 to 69. The demographics and professional characteristics of the participants are shown in Table 2.

### 3.2. Burnout, Depression, and Stressors for All Respondents

The average scores obtained on the scales and subscales and the percentages of different levels of severity obtained by frequency tables are shown in Table 3.

Results showed 100% prevalence of Maslach burnout (56.1% moderate to severe burnout) and 63.1% prevalence of depression.

In our study group, by calculating the average scores obtained on scales and subscales, we obtained medium scores for Maslach emotional exhaustion (24.25 ± 10.03), Maslach personal achievement reduction (23.71 ± 7.92), Maslach burnout (59.92 ± 20.58), and PHQ-9 depression (7.62 ± 5.78). We obtained low scores for Maslach depersonalization (10.96 ± 4.40), CBI personal burnout (47.97 ± 23.39), CBI work-related burnout (44.76 ± 19.35), and CBI client-related burnout (29.19 ± 23.29). Scores for Karasek job factors, job demands, and job control were perceived as being low, with average scores of (19.56 ± 3.00) and (61.44 ± 10.69), while social support was perceived as being high (25.09 ± 3.69).

### 3.3. Burnout, Depression, and Stressors by Category of Professions

The analysis of the scores obtained from different professional categories showed that resident doctors had high levels of Maslach emotional exhaustion, Maslach depersonalization, Maslach personal achievement reduction, and Maslach burnout combined with high Karasek job demands and low Karasek job control compared to the rest of the professional categories. Low scores in all the analyzed categories were reported by professional caregivers (Table 3). The resident doctors had the highest prevalence of high severity in Maslach emotional exhaustion (93.3%), Maslach personal achievement reduction (60%), and Maslach burnout (66.7%), as well as the highest prevalence of severe CBI personal burnout (6.7%) and PHQ-9 depression (26.7%). Resident doctors reported the highest prevalence of high severity in Karasek job demands (53.3%) and the highest prevalence of low severity in Karasek job control (100.0%), Table 3.

### 3.4. The Relation between Profession and Categories and Subcategories of Burnout, Depression, and Occupational Stressors

There were significant differences between professional groups in eight of the subscales, as presented in Table 4.

In the case of the Maslach depersonalization scale, client-related burnout, and social support on the Karasek scale, the scores did not show a significant difference between professions.

Therefore, observing the effect sizes, the profession explained 38% of the scores for Maslach emotional exhaustion, 32.5% for Maslach reduced personal achievement, 32.5% for Maslach burnout, 25% for CBI personal burnout, 19% for CBI work-related burnout, 18% for Karasek job demands, 20% for Karasek job control, and 34% for PHQ-9 depression severity.

The analysis of comparisons of the post hoc one-way ANOVA with independent samples found the following significant differences between the different professional categories, as shown in Table 5.

Noteworthy are the significant differences between resident doctors and specialist doctors in emotional exhaustion, Karasek job control, and depression.

### 3.5. The Relation between Age Groups and Categories and Subcategories of Burnout, Depression, and Occupational Stressors

We analyzed the influence of age on categories and subcategories of burnout, depression, and professional stressors using the following age intervals: 22–30 years, 31–40 years, 41–50 years, 51–60 years, and 61–69 years.

There were significant differences between age groups in eight subscales, as presented in Table 6.

Regarding the effect sizes, the difference between age groups explained 14% of the variation in Maslach emotional exhaustion scores, 11% of the Maslach depersonalization scores, 16% for Maslach personal achievement reduction, 16% for Maslach burnout, 14% for CBI personal burnout, 13% for CBI work-related burnout, 10% for Karasek job control, and 18% for the PHQ-9 depression severity.

The analysis of post hoc one-way ANOVA comparisons with independent samples found significant differences between the age group of 22–30 years and the age group of 41–50 years in scores for Maslach emotional exhaustion, Maslach personal achievement reduction, Maslach burnout, CBI personal burnout, CBI work-related burnout, and PHQ-9 depression severity, as shown in Table 7. There were also differences between the age group of 22–30 years and the age group of 51–60 years in the scores for Maslach reduction in personal achievement, CBI personal burnout, and PHQ-9 depression severity.

### 3.6. The Relation between Professional Experience in the Present Job and the Current Profession on Categories and Subcategories of Burnout, Depression, and Occupational Stressors

The influence of total years of work on the categories and subcategories of burnout, depression, and occupational stressors was analyzed, with years of work experience being grouped into 0–10 years, 11–20 years, 21–30 years, 31–40 years, and 41–45 years.

There were significant differences between age groups in seven subscales, as presented in Table 8.

Regarding the effect sizes, the differences between professional experience groups explain 11% of the variation in Maslach emotional exhaustion scores, 14% of the variation in scores for Maslach personal achievement reduction, 13% for Maslach burnout, 13% for CBI personal burnout, 9.7% for CBI work-related burnout, 11% for Karasek job demands, and 15% for PHQ-9 depression severity.

The analysis of post hoc one-way ANOVA comparisons with independent samples found significant differences between the group with 0–10 years and that with 11–20 years of work experience in scores for Maslach reduction in personal achievements, Maslach burnout, Karasek job demands, and PHQ-9 depression severity, as shown in Table 9. There were also differences between the group with 0–10 years and that with 21–30 years of work experience in the Maslach reduction in personal achievements and PHQ-9 depression severity scores. Regarding PHQ-9 depression severity, there were significant differences between the groups with 0–10 years and 11–40 years of work experience, with higher scores being found in the first group.

### 3.7. The Relation between Maslach Burnout Score and Sex, Age, and Profession

To further assess the predictors of burnout, we built a multiple linear regression model with the natural logarithm of the Maslach burnout score as the dependent variable and sex, age, and profession as independent variables (Table 10). Age and gender were not statistically significantly related to the burnout score. However, the factor of profession remained statistically significant. The resident doctors had significantly higher burnout scores, while nurses and professional caregivers had lower burnout scores compared to infectious disease specialists. A 5-fold cross-validation of this model, repeated 200 times, was performed, and obtained an adjusted R-squared of 0.329 and a root mean square deviation of 0.288.

## 4. Discussion

Prevention and management strategies for mental suffering in healthcare workers appeared as important challenges during the COVID-19 pandemic. Our survey managed to identify the current burnout and depression statuses in professionals working exclusively with a large number of COVID-19 patients and facing a large number of deaths due to COVID-19.

We chose the Maslach scale due to its good psychometric properties: the Cronbach alpha coefficient for exhaustion, cynicism, and effectiveness are, respectively, 88, 90, and 84 (*p* < 0.05), and it has good internal consistency. In addition, the test–retest reliability was good, at about four weeks, with reliability coefficients of 89, 84, and 67 (*p* < 0.01) for exhaustion, cynicism, and effectiveness, respectively [24].

We also decided to use CBI to compare the results with the Maslach burnout score and verify whether personal burnout could be differentiated from that related to the job or clients.

Regarding professional stress factors, we chose the Karasek job factors questionnaire derived from the Karasek Job Content Questionnaire (JCQ) because it was found to be a valid tool for measuring psychosocial pressure in the working environment [25]. The JCQ was developed based on the two dimensions of the ‘requirements-control’ Karasek model [26]. The ‘requirements’ dimension represents organizational duties and constraints, and the ‘control’ dimension describes the job control and control of the employee’s work, which gives one a feeling of autonomy. Later, Karasek and Theorel, 1990, expanded the two-dimensional model and included “social support” from colleagues and superiors [27].

In addition, PHQ-9, accessible to the public in The Diagnostic and Statistical Manual of Mental Disorders (DSM-5), was used because of its 88% sensitivity and specificity for major depression [28].

The present study found a similar prevalence of moderate and severe Maslach burnout (56.10%) compared to the prevalence of burnout reported by Morgantini et al. in 2020, which detected burnout in 51% of 2707 healthcare professionals on the frontline of COVID-19 [29]. The Morgatini study also found a significant relationship between burnout and the inability to perform household activities, the feeling of being forced to carry out activities for which participants had not been trained, and the need to prioritize activities. Burnout was higher among participants in higher-income countries than in middle- and low-income countries [29]. It is also important to underline that healthcare workers may have had their own sick family members, childcare issues, and personal affairs impressing upon them from the outside world, which may have left them feeling pulled between a sense of duty to their patients and their loved ones [30].

Compared with the prevalence of Maslach burnout reported in a similar Romanian hospital one year after the beginning of the COVID-19 pandemic (61.86%) [16], we found a similar prevalence (60.5%) of moderate and high burnout two years after the onset of the pandemic. Maintaining high levels of burnout over time shows that, although there have been periods of relative lulls between pandemic waves at the population level, the pressure on medical personnel remains high.

The assessment of burnout with the CBI scales showed a lower prevalence of burnout compared to the Maslach assessment. Thus, the prevalence of moderate, high, and severe CBI personal burnout, work-related burnout, and client-related burnout was 45.6%, 44.7%, and 22.8%, as presented in Table 3. The lower prevalence of burnout assessed by the CBI scales than that assessed by the Maslach scale may be due to the fact that only some of the Maslach scale items refer to emotional exhaustion, while the other items refer to coping (Maslach depersonalization) and long-term consequences (Maslach reduction in personal achievements)—items that are not included in the CBI scales [19].

Regarding depression, 63.1% of the staff experienced depression (mild, moderate, high, or severe). The prevalence of PHQ-9 depression severity was higher than the prevalence of Maslach burnout on all three subscales (56.10%), but was close to the prevalence of Maslach emotional exhaustion (60.5%). The similar prevalence may be justified by the fact that Maslach’s emotional exhaustion is a common symptom in both burnout and depression, which supports the ICD11 differential diagnosis between the two [3].

This study also aimed to assess professional stressors using the Karasek job factors questionnaire and found that, at the level of the entire study group, 30.7% considered that job demands were high, 82.5% considered they had low job control, and 72.8% thought they had increased social support (Karasek scales). The high Karasek psychological demands associated with low Karasek job control may be cumulative stressors that favor the emergence of emotional exhaustion and the sense of a reduction in personal achievements, as assessed by the Maslach scales. The sense of a reduction in personal achievements may lead to depression, and is usually associated with self-depreciation and the feeling of failure. Increased social support, which was considered to be present in a large portion of the staff, is a protective factor and can explain the low percentage of moderate/increased Maslach depersonalization (33.3%). This means increased mental distance from one’s job or feelings of negativity or cynicism related to one’s job.

Regarding the category of professions, in terms of burnout syndrome subscales and depression, the resident doctors had the highest prevalence compared to the rest of the staff, associated with the highest job demands and lowest job control, as presented in Table 3. This difference was also found in statistical analyses of the effect of profession on categories and subcategories of burnout, depression, and professional stressors (Table 5), which is consistent with other studies comparing experienced physicians and physicians in training (resident doctors) [31]. Furthermore, in the multiple regression analysis, resident doctors continued to have significantly higher burnout scores than infectious disease specialists. These results can be interpreted as being caused by the fact that resident doctors specializing in infectious diseases were included in managing a large number of hospitalized COVID-19 patients under the conditions of a lack of professional experience with severe pathologies and stress associated with the emergent infectious disease. The feeling of reduced personal achievement and the low Karasek job control present in all resident doctors caused distrust in their capacities, avoidance of decision-making, lack of autonomy, low professional satisfaction, and, finally, depression. In the multiple regression analysis, professional caregivers and administrative staff had lower burnout scores compared to infectious disease specialists, albeit the result was statistically significant only for professional caregivers.

The prevalence of moderate and high burnout among infectious disease specialists in our study group was 71.4%, higher than the 45% prevalence reported by the Medscape National Physician Burnout and Suicide Report in 2020 [32].

To observe the dynamics of burnout syndrome, we compared the prevalence of moderate and high Maslach scores one year into the pandemic, as found by Dumea et al., with our observations at two years into the pandemic in Table 11.

It was found that among all the participants, the prevalence of burnout symptoms was higher at two years than at one year after the onset of the COVID-19 pandemic, explained by the continuous exposure to the same risk factors.

We observed significantly higher scores in the age group of 22–30 years compared to the age group of 41–50 years and older (Table 6 and Table 7) on all scales except Maslach depersonalization, CBI client-related burnout, and Karasek job factors. We did not find significant differences in job factors (job demands, job control, and social support), which shows that there are other psychological factors that cause burnout and depression in young people. A reduced identity status associated with reduced work engagement may be the explanation [33]. We found higher scores for those with 0–10 years of work experience compared to those with more work experience for Maslach reduction in personal achievements, Maslach burnout, Karasek job demands, and PHQ-9 depression severity (Table 8 and Table 9). These data support the idea that less work experience is more associated with burnout and depression at work, possibly due to less accommodation to the job’s demands. We concluded that young medical personnel (including resident doctors) are more vulnerable than seniors to burnout symptoms and depression.

Our results can be considered comparable to Medscape National Physician Burnout and Suicide Report 2020, which showed that the average burnout prevalence reached 45% among physicians working in the field of infectious disease. The prevalence of burnout can even exceed 50% in doctors if both practicing and training doctors (resident doctors) are considered. The most important causes found were: too many bureaucratic tasks (55%); too many hours of work (33%); a lack of respect from administrative staff, colleagues, and management (32%); increased computer activity (30%); insufficient compensation (29%); lack of autonomy and control (24%); a feeling of meaninglessness (22%); a lack of respect from patients (17%); and other (7%) [32]. Cross-sectional studies have associated physician burnout with decreased productivity, job dissatisfaction, and even intent to leave one’s current job for reasons other than retirement, with large consequences for the physician workforce and healthcare system costs [31]. A study published in February 2020, before the pandemic, was already forecasting physician workforce shortages in USA for 2030. Improving the quality of care, increasing access to care, and controlling healthcare costs depend on the adequate availability of healthcare providers [34].

In addition to burnout, doctors face dissatisfaction with the balance between life and work, dissatisfaction regarding career choices, major depressive disorder (MDD), substance use and abuse, and unacceptable suicide rates. Worryingly, physician depression and suicide prevention are relatively ignored, in part fueled by the fact that burnout and MDD have overlapping symptoms and clinical features [35]. Every year, more than 400 doctors commit suicide, probably due to increased burnout and depression. Most cases are due to long hours of work, substantial educational debt, and a culture in which ‘no mistakes are admitted;. The sense of guilt and isolation resulting from medical errors or poor results can lead to emotional wounds to the doctor, the so-called ‘second victim’ syndrome, which contributes to and is a consequence of exhaustion [36,37]. This is the reason why we chose to examine the levels of burnout and depression among doctors.

Regarding nurses, we found a 40% prevalence of moderate and high Maslach emotional exhaustion, which is higher than the 11.23% prevalence found in the first meta-analysis conducted in early 2020, which involved 45,539 nurses from 49 countries in several specialties [38]. In the 2020 study, significant differences were observed between geographical regions, specialties, and the type of measurement of exhaustion used. The Sub-Saharan Africa region had the highest prevalence rate of symptoms of exhaustion, while the regions of Europe and Central Asia had the lowest rate. Pediatric nurses had the highest prevalence of burnout symptoms out of all specialties, while geriatric nurses had the lowest.

In our study, in the univariate analysis, there were no significant differences found between experienced physicians and nurses for any of the analyzed scales. In the multiple regression analysis, we found that nurses experienced less burnout compared to infectious disease specialists. In one study, nurses were shown to be the most affected working category, with higher levels of distress and fear of uncontrollable virus spread and lower levels of trust in guidelines compared to physicians [39]. However, a study published in 2020 that assessed burnout among medical staff in Spain during the first period of the 2019 COVID-19 pandemic had similar findings to our research, with doctors having a higher level of burnout than nurses [40].

A study performed in June 2020 on healthcare workers in the UK that assessed burnout levels, anxiety, depression, and distress due to the COVID-19 pandemic showed that burnout was present in 79% of the participants, with 76% reporting high levels of stress. Most of them were young and female, had transferred from other areas of activity to work with COVID-19-infected patients, worried about access to protective equipment, and had a previous history of depression. A total of 77% of those who reported high levels of stress did not receive social support, saying they either did not feel they needed support, did not have time, that support was not relevant, or did not want their colleagues to know they struggled with stress [41].

A study published in 2020 proved the moderating effect of social support on the relationship between burnout and anxiety symptoms [42].

Regarding depression, we found a prevalence of 63.1% of mild, moderate, high, and severe depression using the PHQ-9 scale, which is higher than the 50.4% prevalence found in a study conducted in January–February 2020 in China. The same scale was used for 1257 participants, including health workers from 34 hospitals with dedicated COVID-19 wards [43]. Our results are quite similar to those obtained with the Depression Anxiety Stress Scales (DASS-21) in a study conducted in Turkey in March 2020 on 442 participants who were on the front line and caring for patients with COVID-19, which showed that 64.7% of participants had symptoms of depression, 51.6% had anxiety, and 41.2% had stress [44]. Regarding the evolution of depression symptoms during the two years of the COVID-19 pandemic in Poland, a cross-sectional study covering four waves was published in 2022 and showed that the first three waves had more impact compared to the fourth wave [45].

In a meta-analysis that included 30 eligible 2020 studies, the total prevalence of depression was 37% (95% CI: 29–45%) [46], whereas, in our study, a prevalence of 30.6% of moderate–severe depression was reported.

### 4.1. Limitations and Strengths

The low response rate (10.83%) from the total staff is the main limitation of the study; this can be explained by the large number of medical staff who took summer holidays between waves 5 and 6 of the COVID-19 pandemic and by the low willingness to participate in the study due to WHO ‘pandemic fatigue’ [47]. On the other hand, the phasic course of the infection, with waves of increase in the number of COVID-19 cases alternated to periods of lull, could also have increased the uncertainty and the feelings of not being in control of the situation, which may be associated with the low response rate in our study. The exact mechanism of non-response and how the selection bias would modify the results of a survey are classical issues with this design. One possibility might be that employees who suffered from burnout were more likely to have participated in the study. If this is true, then the prevalence of burnout would be overestimated. Another possibility might be that depersonalization within burnout might diminish the response rate of those with burnout to our survey. In this case, the prevalence of burnout would be underestimated. In any case, this would induce a selection bias that could affect the internal validity of the study, as well as the external validity of the study, by its generalization to the target population.

We did not determine the level of burnout and depression before the pandemic in the same group of healthcare workers, so we cannot compare the evolution of prevalence over time. It would be useful to continue the study, using the same scales at regular intervals, to see to what extent the symptoms of burnout and depression are maintained. Considering the risk of delayed psychiatric issues, intervention should not be limited to a certain amount of time and should be offered to healthcare workers even after the crisis period. The observational nature of the study design cannot determine causal relationships between the professional groups, age groups, professional experience, and depression or burnout. The results are more likely to be generalized to similar hospitals in Romania. It is reasonable to consider that similar problems have been encountered worldwide, and the burden of the pandemic has similarly generated burnout and depression.

Our study demonstrates data regarding the psychological aspects of the COVID-19 pandemic’s consequences for healthcare workers’ mental health in a first-line hospital dedicated exclusively to COVID-19 that managed many COVID-19 patients in the two years of the pandemic. Another strength of this study is the use of four different scales to assess the psychological response to the pandemic in healthcare workers. Moreover, two scales for burnout were employed to broaden the picture of the newly WHO-recognized mental health problem of burnout as a factor influencing health status.

### 4.2. Relevance to Clinical Practice and Public Health

Though data were not included in the present study, the investigation was performed with the final aim of recommending specialized psychiatric consultations.

There are a number of concrete organizational strategies useful for minimizing psychological risks among healthcare workers. Introducing priority partnerships between preventive and clinical health professions (infectious disease specialists, resident doctors, nurses, professional caregivers, psychologists, psychiatrists, and social workers) and other professionals engaged in institutional activities to safeguard the community and to improve public health might pave the way for interdisciplinary growth [48]. Giving workers access to counseling or therapy services is one way that healthcare institutions may assist in improving their mental health. This might take the form of a community partnership with mental health care providers or an employee assistance program (EAP) that offers private counseling services. This can include workshops on mindfulness, stress management, and coping strategies. Encouraging open communication by fostering an environment where healthcare professionals feel encouraged to talk about their feelings and mental health can lessen the stigma associated with asking for help. Building trust and establishing open channels of communication may be facilitated by encouraging frequent check-ins with management and coworkers. Healthcare institutions should employ workload management strategies, since high workloads and lengthy working hours are risk factors for burnout and other psychological problems. To achieve this workload management, initiatives could include team-based care, task delegation, and flex-time scheduling. Healthcare organizations may put rules in place to make sure that employees have enough vacation time and are encouraged to take frequent breaks during the workday.

## 5. Conclusions

The COVID-19 pandemic continues to have consequences on mental health among healthcare workers involved in the management of COVID-19 patients who require hospitalization.

Results showed a 100% prevalence of burnout (56.1% moderate and severe burnout) and a 63.1% prevalence of depression. The highest prevalence of burnout scores (Maslach and CBI), depression severity (PHQ-9), and job demands (Karasek) was found among young doctors in training (infectious diseases resident doctors). The 22- to 30-year-old age group and the group with less than ten years of professional experience had a significantly higher prevalence of burnout and depression than older employees and employees with longer professional experience. The COVID-19 pandemic continues to have a significant impact on the mental health of healthcare workers.

Further studies are needed to identify burnout and find solutions for first-line medical staff to prevent and reduce burnout and depression, thus preventing the professional crisis in the healthcare system due to prolonged medical leaves and resignation of healthcare workers.

## Figures and Tables

**Table 1:** Cut-off values for the scales/subscales.

Scale	Subscale	Score Severity Level				
		Without	Low	Medium	High	Severe
Maslach Burnout Inventory [1]	Emotional exhaustion	1–8	9–18	19–27	28–45	
	Depersonalization	1–6	6–12	13–18	19–30	
	Personal achievement reduction	1–9	10–20	21–30	31–50	
	Burnout	0–24	25–50	51–75	76–125	
Copenhagen Burnout Inventory [19,23]	Personal burnout		0–49 *	50–74	75–99	100
	Work-related burnout		0–49 *	50–74	75–99	100
	Client-related burnout		0–49 *	50–74	75–99	100
Karasek job factors questionnaire [21]	Job demands		≤20		>20	
	Job control		<71		≥71	
	Social support		<24		≥24	
The Adult Depression Severity scale adapted from the PHQ-9 [22]	Depression	0–4	5–9	10–14	15–19	20–27

*, no/low severity level; PHQ-9, Patient Health Questionnaire-9.

**Table 2:** Demographics and professional characteristics of respondents.

Characteristic	N = 114
Age (years), mean ± SD	41.38 ± 10.60
women	42.05 ± 10.31
men	38.20 ± 11.64
Sex, n (%)	
women	94 (82.5)
men	20 (17.5)
Profession, n (%)	
infectious disease specialists	33 (28.95)
resident doctors	15 (13.16)
infectious disease nurses	34 (29.82)
pharmacists	3 (2.63)
radiologists	2 (1.75)
professional caregivers	11 (9.65)
physical therapists	1 (0.88)
social workers	1 (0.88)
psychologists	1 (0.88)
administrative personnel	13 (11.40)
Management position, n (%)	
leadership position *	10 (8.8)
no leadership position	104 (91.2)
Years of work in the current profession, mean ± SD	17.96 ± 11.99
women	18.83 ± 11.66
men	13.90 ± 13.03

SD, standard deviation; *, 8 doctors and 2 administrative personnel.

**Table 3:** Burnout, depression, and Karasek stressors by personnel category and percentage of people by severity level.

		Infectious Disease Specialists and Radiologists (N = 35)	Resident Doctors (N = 15)	Nurses + Physical Therapists (N = 35)	Professional Caregivers (N = 11)	Pharmacists (N = 3)	Administrative Personnel (N = 13)	Social Workers and Psychologists (N = 2)	Total (N = 114)
Maslach emotional exhaustion	Mean ± SD (Interpretation *)	26.31 ± 8.08 (Medium)	35.87 ± 4.91 (High)	20.43 ± 9.99 (Medium)	12.81 ± 4.21 (Low)	19.00 ± 8.18 (Medium)	25.38 ± 7.87 (Medium)	31.50 ± 6.36 (High)	24.25 ± 10.03 (Medium)
Severity categories	Low (9–18), n (%)	9 (25.7)	0.0	21 (60.0)	9 (81.8)	2 (66.7)	4 (30.8)	0.0	45 (39.5)
	Medium (19–27), n (%)	10 (28.6)	1 (6.7)	7 (20.0)	2 (18.2)	0.0	1 (7.7)	1 (50.0)	22 (19.3)
	High (28–45), n (%)	16 (45.7)	14 (93.3)	7 (20.0)	0.0	1 (33.3)	8 (61.5)	1 (50.0)	47 (41.2)
Maslach depersonalization	Mean ± SD (Interpretation *)	11.57 ± 4.63 (Medium)	13.00 ± 2.69 (High)	10.77 ± 4.88 (Medium)	7.54 ± 1.57 (Low)	9.33 ± 2.52 (Low)	10.77 ± 4.58 (Low)	10.50 ± 6.36 (Low)	10.96 ± 4.40 (Low)
Severity categories	Low (6–12), n (%)	22 (62.9)	7 (46.7)	24 (68.6)	11 (100.0)	3 (100.0)	8 (61.5)	1 (50.0)	76 (66.7)
	Medium (13–18), n (%)	10 (28.6)	8 (53.3)	8 (22.9)	0.0	0.0	4 (30.8)	1 (50.0)	31 (27.2)
	High (19–30), n (%)	3 (8.6)	0.0	3 (8.6)	0.0	0.0	1 (7.7)	0.0	7 (6.1)
Maslach personal achievement reduction	Mean ± SD (Interpretation *)	25.51 ± 6.46 (Medium)	33.47 ± 6.70 (High)	20.51 ± 7.52 (Medium)	18.91 ± 4.61 (Low)	19.33 ± 4.04 (Low)	21.15 ± 6.18 (Medium)	24.50 ± 10.61 (Medium)	23.71 ± 7.92 (Medium)
Severity categories	Low (10–20), n (%)	8 (22.9)	0.0	25 (71.4)	7 (63.6)	2 (66.7)	7 (53.8)	1 (50.0)	50 (43.9)
	Medium (21–30), n (%)	21 (60.0)	6 (40.0)	4 (11.4)	4 (36.4)	1 (33.3)	5 (38.5)	0.0	41 (36.0)
	High (31–50), n (%)	6 (17.1)	9 (60.0)	6 (17.1)	0.0	0.0	1 (7.7)	1 (50.0)	23 (20.1)
Maslach burnout	Mean ± SD (Interpretation *)	63.40 ± 17.27 (Medium)	82.33 ± 12.50 (High)	51.71 ± 21.40 (Medium)	39.27 ± 8.69 (Low)	47.66 ± 10.21 (Low)	57.31 ± 15.65 (Medium)	66.50 ± 23.33 (Medium)	59.92 ± 20.58 (Medium)
Severity categories	Low (25–50), n (%)	10 (28.6)	0.0	23 (65.7)	10 (90.9)	1 (33.3)	5 (38.5)	1 (50.0)	50 (43.9)
	Medium (21–75), n (%)	17 (48.5)	5 (33.3)	6 (17.2)	1 (9.1)	2 (66.7)	7 (53.8)	0.0	38 (33.3)
	High (76–125), n (%)	8 (22.9)	10 (66.7)	6 (17.1)	0.0	0.0	1 (7.7)	1 (50.0)	26 (22.8)
CBI personal burnout	Mean ± SD (Interpretation *)	51.53 ± 20.43 (Medium)	71.10 ± 15.78 (Medium)	42.16 ± 25.52 (Low)	25.64 ± 16.40 (Low)	37.50 ± 4.50 (Low)	48.04 ± 15.91 (Low)	52.00 ± 26.87 (Medium)	47.97 ± 23.39 (Low)
Severity categories	Low (0–49), n (%)	17 (48.6)	1 (6.7)	24 (68.6)	10 (90.9)	3 (100.0)	6 (46.2)	1 (50.0)	62 (54.4)
	Medium (50–74), n (%)	12 (34.3)	8 (53.3)	5 (14.3)	1 (9.1)	0.0	6 (46.2)	1 (50.0)	33 (28.9)
	High (75–99), n (%)	6 (17.1)	5 (33.3)	5 (14.3)	0.0	0.0	1 (7.7)	0.0	17 (14.9)
	Severe (100), n (%)	0.0	1 (6.7)	1 (2.9)	0.0	0.0	0.0	0.0	2 (1.8)
CBI work-related burnout	Mean ± SD (Interpretation *)	47.50 ± 16.92 (Low)	62.10 ± 12.72 (Medium)	38.96 ± 20.72 (Low)	34.91 ± 15.34 (Low)	27.50 ± 13.54 (Low)	43.65 ± 20.00 (Low)	55.50 ± 7.78 (Medium)	44.76 ± 19.35 (Low)
Severity categories	Low (0–49), n (%)	16 (45.7)	2 (13.3)	26 (74.3)	9 (81.8)	3 (100.0)	7 (53.8)	0.0	63 (55.3)
	Medium (50–74), n (%)	17 (48.6)	11 (73.3)	6 (17.1)	2 (18.2)	0.0	6 (46.2)	2 (100.0)	44 (38.6)
	High (75–99), n (%)	2 (5.7)	2 (13.3)	3 (8.6)	0.0	0.0	0.0	0.0	7 (6.1)
	Severe (100), n (%)	0.0	0.0	0.0	0.0	0.0	0.0	0.0	0.0
CBI client-related burnout	Mean ± SD (Interpretation *)	34.10 ± 23.86 (Low)	36.80 ± 29.20 (Low)	22.71 ± 20.99 (Low)	25.32 ± 15.77 (Low)	8.33 ± 8.50 (Low)	28.50 ± 21.98 (Low)	56.50 ± 20.51 (Low)	29.19 ± 23.29 (Low)
Severity categories	Low (0–49), n (%)	25 (71.4)	9 (60.0)	30 (85.7)	10 (90.9)	3 (100.0)	10 (76.9)	1 (50.0)	88 (77.2)
	Medium (50–74), n (%)	7 (20.0)	4 (26.7)	4 (11.4)	1 (9.1)	0.0	3 (23.1)	1 (50.0)	20 (17.5)
	High (75–99), n (%)	3 (8.6)	2 (13.3)	1 (2.9)	0.0	0.0	0.0	0.0	6 (5.3)
	Severe (100), n (%)	0.0	0.0	0.0	0.0	0.0	0.0	0.0	0.0
Depression severity (PHQ-9)	Mean ± SD (Interpretation *)	8.80 ± 4.30 (Low)	14.33 ± 5.86 (Medium)	5.77 ± 5.78 (Low)	1.54 ± 2.11 (Absent)	4.66 ± 2.08 (Low)	7.61 ± 3.95 (Medium)	7.00 ± 2.83 (Medium)	7.62 ± 5.78 (Medium)
Severity categories	Absent (0–4), n (%)	7 (20.0)	0.0	21 (60.0)	9 (81.8)	2 (66.7)	3 (23.1)	0.0	42 (36.8)
	Mild (5–9), n (%)	16 (45.7)	4 (26.7)	7 (20.0)	2 (18.2)	1 (33.3)	5 (38.5)	2 (100.0)	37 (32.5)
	Moderate (10–14), n (%)	8 (22.9)	5 (33.3)	2 (5.7)	0.0	0.0	5 (38.5)	0.0	20 (17.5)
	High (15–19), n (%)	3 (8.6)	2 (13.3)	3 (8.6)	0.0	0.0	0.0	0.0	8 (7.0)
	Severe (20–27), n (%)	1 (2.9)	4 (26.7)	2 (5.7)	0.0	0.0	0.0	0.0	7 (6.1)
Karasek job demands	Mean ± SD (Interpretation *)	19.71 ± 2.53 (Low)	22.00 ± 3.87 (High)	19.26 ± 2.67 (Low)	16.82 ± 2.64 (Low)	18.00 ± 2.64 (Low)	19.77 ± 2.62 (Low)	20.00 ± 0.00 (Low)	19.56 ± 3.00 (Low)
Severity categories	Low (≤20), n (%)	23 (65.7)	7 (46.7)	25 (71.4)	(11) 100.0	3 (100.0)	8 (61.5)	2 (100.0)	79 (69.3)
	High (>20), n (%)	12 (34.3)	8 (53.3)	10 (28.6)	0.0	0.0	5 (38.5)	0.0	35 (30.7)
Karasek job control	Mean ± SD (Interpretation *)	66.86 ± 9.90 (Low)	54.53 ± 10.68 (Low)	61.25 ± 10.33 (Low)	52.91 ± 10.21 (Low)	60.66 ± 1.15 (Low)	82.31 ± 7.82 (High)	64.00 ± 0.00 (Low)	61.44 ± 10.69 (Low)
Severity categories	Low (<71), n (%)	23 (65.7)	15 (100.0)	29 (82.9)	(11) 100.0	3 (100.0)	11 (84.6)	2 (100.0)	94 (82.5)
	High (≥71), n (%)	12 (34.3)	0.0	6 (17.1)	0.0	0.0	2 (15.4)	0.0	20 (17.5)
Karasek social support	Mean ± SD (Interpretation *)	23.29 ± 3.19 (Low)	24.80 ± 4.60 (High)	25.86 ± 3.93 (High)	25.36 ± 3.52 (High)	25.00 ± 3.60 (High)	25.92 ± 3.23 (High)	21.00 ± 2.83 (Low)	25.09 ± 3.69 (High)
Severity categories	Low (<24), n (%)	13 (37.1)	3 (20.0)	6 (17.1)	4 (36.4)	1 (33.3)	2 (15.4)	2 (100.0)	31 (27.2)
	High (≥24), n (%)	22 (62.9)	12 (80.0)	29 (82.9)	7 (63.6)	2 (66.7)	11 (84.6)	0.0	83 (72.8)

CBI, Copenhagen burnout inventory; PHQ-9, Patient Health Questionnaire-9; SD, standard deviation; *, interpretation refers to the clinical category of the average score.

**Table 4:** Burnout, depression, and occupational stressors subscales comparison regarding professional groups *.

Scale	F_6,107_^#^	*p*-Value	ƞ^2^
Maslach emotional exhaustion	10.946	<0.001	0.38
Maslach reduction in personal achievements	8.552	<0.001	0.325
Maslach burnout	8.554	<0.001	0.325
CBI personal burnout	5.969	<0.001	0.25
CBI work-related burnout	4.259	<0.001	0.19
Karasek job demands	3.932	<0.001	0.18
Karasek job control	4.431	<0.001	0.20
Depression severity (PHQ-9)	127	<0.001	0.34

CBI, Copenhagen burnout inventory; PHQ-9, Patient Health Questionnaire-9; *, professional groups: infectious diseases specialists and radiologists, resident doctors, nurses and physical therapists, professional caregivers, pharmacists, administrative personnel, social workers and psychologists; ^#^, ANOVA F statistic with degrees of freedom; ƞ^2^, effect size.

**Table 5:** Multiple comparisons of means between different professional categories.

Dependent Variable	Profession	Mean Difference	*p*-Value
Maslach emotional exhaustion	Resident doctors vs. infectious disease specialists and radiologists	9.55	0.031
	Resident doctors vs. professional caregivers	15.44	<0.001
	Infectious disease specialists and radiologists vs. nurses and physical therapists	13.50	0.002
	Administrative staff vs. professional caregivers	12.57	0.034
Maslach personal achievement reduction	Resident doctors vs. infectious disease specialists and radiologists	7.95	0.028
	Resident doctors vs. nurses and physical therapists	12.95	<0.001
	Resident doctors vs. administrative personnel	12.31	0.001
Maslach burnout	Resident doctors vs. professional caregivers	43.06	<0.001
	Resident doctors vs. nurses and physical therapists	30.62	<0.001
	Resident doctors vs. administrative personnel	25.03	0.032
	Infectious disease specialists and radiologists vs. professional caregivers	24.13	0.018
CBI personal burnout	Resident doctors vs. professional caregivers	45.46	<0.001
	Resident doctors vs. nurses and physical therapists	28.94	0.004
CBI work-related burnout	Resident doctors vs. professional caregivers	27.19	0.029
	Resident doctors vs. nurses and physical therapists	23.14	0.011
Karasek job demands	Resident doctors vs. professional caregivers	5.18	0.003
Karasek job control	Resident doctors vs. infectious disease specialists and radiologists	−12.32	0.016
PHQ-9 depression severity	Resident doctors vs. infectious disease specialists and radiologists	5.53	0.040
	Resident doctors vs. nurses and physical therapists	8.56	<0.001
	Resident doctors vs. administrative personnel	6.72	0.044
	Infectious disease specialists and radiologists vs. professional caregivers	7.25	0.007

CBI, Copenhagen burnout inventory; PHQ-9, Patient Health Questionnaire-9.

**Table 6:** Burnout, depression, and occupational stressors subscales comparison regarding age groups.

Scale	F_4,109_^#^	*p*-Value	ƞ^2^
Maslach emotional exhaustion	4.558	0.02	0.14
Maslach depersonalization	3.342	0.013	0.11
Maslach personal achievement reduction	5.219	0.001	0.16
Maslach burnout	5.343	0.001	0.16
CBI personal burnout	4.304	0.003	0.14
CBI work-related burnout	4.030	0.004	0.13
Karasek job control	3.122	0.018	0.10
PHQ-9 depression severity	5.843	*p* < 0.01	0.18.

CBI, Copenhagen burnout inventory; PHQ-9, Patient Health Questionnaire-9; ^#^, ANOVA F statistic with degrees of freedom; ƞ^2^, effect size.

**Table 7:** Multiple comparisons of means between different age groups.

Dependent Variable	Age Intervals	Mean Difference	*p*-Value
Maslach emotional exhaustion	22–30 years vs. 41–50 years	9.80	0.003
Maslach reduction in personal achievements	22–30 years vs. 41–50 years	7.85	0.002
	22–30 years vs. 51–60 years	7.05	0.047
Maslach burnout	22–30 years vs. 41–50 years	20.97	0.002
CBI personal burnout	22–30 years vs. 41–50 years	20.93	0.010
	22–30 years vs. 51–60 years	21.53	0.040
CBI work-related burnout	22–30 years vs. 41–50 years	17.05	0.012
PHQ-9 depression severity	22–30 years vs. 41–50 years	6.20	0.001
	22–30 years vs. 51–60 years	5.34	0.032

CBI, Copenhagen burnout inventory; PHQ-9, Patient Health Questionnaire-9.

**Table 8:** Burnout, depression, and occupational stressors subscales comparison regarding total years of work at the present job.

Scale	F_4,109_ *^#^*	*p*-Value	ƞ^2^
Maslach emotional exhaustion	3.371	0.01	0.11
Maslach personal achievement reduction	4.566	0.002	0.14
Maslach burnout	4.143	0.004	0.13
CBI personal burnout	4.024	0.004	0.13
CBI work-related burnout	2.930	0.024	0.097
Karasek job demands	3.338	0.013	0.110
PHQ-9 depression severity	4.850	0.001	0.15.

CBI, Copenhagen burnout inventory; PHQ-9, Patient Health Questionnaire-9; ^#^, ANOVA F statistic with degrees of freedom; ƞ^2^, effect size.

**Table 9:** Multiple comparisons of means between different groups of professional experience.

Dependent Variable	Years of Work	Mean Difference	*p*-Value
Maslach reduction in personal achievements	0–10 years vs. 11–20 years	6.10	0.035
	0–10 years vs. 21–30 years	6.40	0.021
Maslach burnout	0–10 years vs. 21–30 years	15.60	0.038
Karasek job demands	0–10 years vs. 11–20 years	2.31	0.042
PHQ-9 depression severity	0–10 years vs. 11–20 years	4.66	0.023
	0–10 years vs. 21–30 years	4.42	0.033
	0–10 years vs. 31–40 years	5.26	0.037

PHQ-9, Patient Health Questionnaire-9.

**Table 10:** Multiple linear regression predicting the natural logarithm of Maslach burnout score based on sex, age, and profession, with robust and bootstrapped bias-corrected and accelerated confidence intervals.

	B	95% CI Robust *	95% CI Bootstrap **	*p*-Value
Sex (male vs. female)	−0.06	−0.21–0.08	−0.19–0.08	0.395
Age (years)	0	−0.01–0.001	−0.009–0.002	0.245
Profession				
Resident doctors vs. infectious disease specialists	0.25	0.04–0.45	0.071–0.42	0.019
Nurses vs. infectious disease specialists	−0.25	−0.38–−0.12	−0.37–−0.10	<0.001
Professional caregivers vs. infectious disease specialists	−0.48	−0.67–−0.28	−0.64–−0.30	<0.001
Administrative staff vs. infectious disease specialists	−0.09	−0.27–0.08	−0.26–0.07	0.297

CI, confidence interval; *, robust confidence intervals based on sandwich estimators; **, bootstrapped biased corrected and accelerated confidence intervals.

**Table 11:** Comparisons of the Maslach syndrome scale and subscales between our study and the study of Dumea et al.

Moderate and High Maslach Scores	Our Study—2 Years of Pandemic (N = 114)	Dumea et al. [16]—1 Year of Pandemic (N = 186)
Emotional exhaustion	74.3%	37.63%
Depersonalization	37.2%	34.6%
Reduction personal achievements	77.1%	65.4%
Burnout	71.4%	69.2%

## Data Availability

Data are available upon request due to restrictions, e.g., privacy-related or ethical.

## References

[1] Maslach C., Jackson S.E. (1981). The measurement of experienced burnout. J. Organ. Behav..

[2] Maslach C., Leiter M.P. (2016). Understanding the burnout experience: Recent research and its implications for psychiatry. World Psychiatry.

[3] ICD-11 for Mortality and Morbidity Statistics (Version: 02/2022). https://icd.who.int/browse11/l-/en#/http%3a%2f%2fid.who.int%2ficd%2fentity%2f129180281.

[4] Toppinen-Tanner S., Ojajärvi A., Väänänen A., Kalimo R., Jäppinen P. (2005). Burnout as a predictor of medically certified sick-leave absences and their diagnosed causes. Behav. Med..

[5] Peterson U., Demerouti E., Bergström G., Samuelsson M., Åsberg M., Nygren Å. (2008). Burnout and physical and mental health among Swedish healthcare workers. J. Adv. Nurs..

[6] (2015). European Working Conditions Surveys (EWCS). https://www.eurofound.europa.eu/publications/report/2016/working-conditions/sixth-european-working-conditions-survey-overview-report.

[7] Working Conditions in the Time of COVID-19: Implications for the Future. https://www.eurofound.europa.eu/publications/report/2022/working-conditions-in-the-time-of-covid-19-implications-for-the-future.

[8] Shanafelt T.D., Boone S., Tan L., Dyrbye L.N., Sotile W., Satele D., West C.P., Sloan J., Oreskovich M.R. (2012). Burnout and satisfaction with work-life balance among US physicians relative to the general US population. Arch. Intern. Med..

[9] De Hert S. (2020). Burnout in Healthcare Workers: Prevalence, Impact and Preventative Strategies. Local Reg. Anesth..

[10] Bradley M., Chahar P. (2020). Burnout of Healthcare Providers during COVID-19. Clevel. Clin. J. Med..

[11] Shanafelt T., Ripp J., Trockel M. (2020). Understanding and addressing sources of anxiety among health care professionals during the COVID-19 pandemic. JAMA.

[12] Rama S.A. (2022). Addressing Burnout, Anxiety and Self-Care in the Age of COVID-19, Diagnostic Imaging. https://www.diagnosticimaging.com/view/addressing-burnout-anxiety-and-self-care-in-the-age-of-COVID-19.

[13] World Health Organization (2020). Mental Health and Psychosocial Considerations during the COVID-19 Outbreak. https://apps.who.int/iris/bitstream/handle/10665/331490/WHO-2019-nCoV%20-MentalHealth-2020.1-eng.pdf?sequence=1.

[14] Society of Critical Care Medicine (2020). Clinicians Report High Stress in COVID-19 Response. SCCM COVID-19 Rapid-Cycle Survey 2 Report. https://sccm.org/Blog/May-2020/SCCM-COVID19-Rapid-Cycle-Survey-2-Report.

[15] Our World in Data (2022). Romania: Coronavirus Pandemic Country Profile. https://ourworldindata.org/corona-virus/country/romania#daily-confirmed-deaths-how-do-they-compare-to-other-countries.

[16] Dumea E., Efrim N.D., Petcu A., Anghel L., Puscasu C.G. (2022). Burnout Syndrome in Personnel of an Infectious Diseases Hospital, One Year after the Outbreak of the COVID-19 Pandemic. BRAIN Broad Res. Artif. Intell. Neurosci..

[17] Maslach C., Jackson S.E., Leiter M.P., Schaufeli W.B., Schwab R.L. (1986). Maslach Burnout Inventory.

[18] Maslach C., Jackson S.E. (1984). Burnout in organizational settings. Applied Social Psychology Annual: Applications in Organizational Settings.

[19] Kristensen T.S., Borritz M., Villadsen E., Christensen K.B. (2005). The Copenhagen Burnout Inventory: A new tool for the assessment of burnout. Work Stress.

[20] Schaufeli W.B., Greenglass E.R. (2001). Introduction to Special Issue on Burnout and Health. Psychol. Health.

[21] Karasek R., Brisson C., Kawakami N., Houtman I., Bongers P., Amick B. (1998). The Job Questionnaire (JCQ). An Instrument for Internationally Comparative Assessments of Psychological Job Characteristics. J. Occup. Health Psychol..

[22] APA_DSM5_Severity-Measure-For-Depression-Adult. https://www.psychiatry.org/File%20Library/Psychiatrists/Practice/DSM/APA_DSM5_Severity-Measure-For-Depression-Adult.pdf.

[23] Creedy D.K., Sidebotham M., Gamble J., Pallant J., Fenwick J. (2017). Prevalence of burnout, depression, anxiety and stress in Australian midwives: A crosssectional survey. BMC Pregnancy Childbirth.

[24] Rostami Z., Mohammad R.A., Schaufeli W.B., Ahmadi A.S., Sadeg A.M. (2014). The Psychometric Characteristics of Maslach Burnout Inventory Student Survey: A Study Students of Isfahan University. Zahedan J. Res. Med. Sci..

[25] Saif-ur-Rehman, Amran R., Bandar A. (2011). The psychometric impacts of Karasek’s demands and control scale on employees’ job dissatisfaction. Afr. J. Bus. Manag..

[26] Karasek R.A. (1979). Job demands, job decision latitude and mental strain: Implications for job redesign. Adm. Saence Q..

[27] Karasek R., Theorell T. (1990). Healthy Work: Stress, Productivity, and the Reconstruction of Working Life.

[28] Kroenke K., Spitzer R.L., Williams J.B.W. (2001). The PHQ-9 Validity of a Brief Depression Severity Measure. J. Gen. Intern. Med..

[29] Morgantini L.A., Naha U., Wang H., Francavilla S., Acar Ö., Flores J.M., Vigneswaran H.T. (2020). Factors contributing to healthcare professional burnout during the COVID-19 pandemic: A Rapid Turnaround Global Survey. PLoS ONE.

[30] Huremović D. (2019). Psychiatry of Pandemics. A Mental Health Response to Infection Outbreak.

[31] West C.P., Dyrbye L.N., Shanafelt T.D. (2018). Physician burnout: Contributors, consequences and solutions. J. Intern. Med..

[32] (2020). Medscape National Physician Burnout & Suicide Report. https://www.medscape.com/slideshow/2020-lifestyle-burnout-6012460.

[33] Luyckx K., Duriez B., Klimstra T., De Witte H. (2010). Identity statuses in young adult employees: Prospective relations with work engagement and burnout. J. Vocat. Behav..

[34] Zhang X., Lin D., Pforsich H., Lin V.W. (2020). Physician workforce in the United States of America: Forecasting nationwide shortages. Hum. Resour. Health.

[35] Oquendo M.A., Bernstein C.A., Mayer L.E. (2019). A key differential diagnosis for physicians—Major depression or burnout?. JAMA Psychiatry.

[36] Stehman C.R., Testo Z., Gershaw R.S., Kellogg A.R. (2019). Burnout, drop out, suicide: Physician loss in emergency medicine, Part I. West. J. Emerg. Med..

[37] Watkins A., Rothfeld M., Rashbaum W.K., Rosenthal B.M., Top E.R. Doctor who Treated Virus Patients Dies by Suicide. The New York Times.

[38] Woo T., Ho R., Tang A., Tam W. (2020). Global prevalence of burnout symptoms among nurses: A systematic review and metaanalysis. J. Psychiatr. Res..

[39] Nissan D., Weiss G., Siman-Tov M., Spitz A., Bodas M., Shenhar G., Adini B. (2021). Differences in Levels of Psychological Distress, Perceived Safety, Trust, and Efficacy amongst Hospital Personnel during the COVID-19 Pandemic. Res. Nurs. Health.

[40] Ruiz-Fernández M.D., Ramos-Pichardo J.D., Ibáñez-Masero O., Cabrera-Troya J., Carmona-Rega M.I., Ortega-Galán A.M. (2020). Compassion fatigue, burnout, compassion satisfaction and perceived stress in healthcare professionals during the COVID-19 health crisis in Spain. J. Clin. Nurs..

[41] Ferry A., Wereski R., Strachan F.E., Mills N.L. (2021). Predictors of healthcare worker burnout during the COVID-19 pandemic. QJM Int. J. Med..

[42] Zhang H., Ye Z., Tang L., Zou P., Du C., Jing Shao J., Wang X., Chen D., Qiao G., Mu M.Y. (2020). Anxiety symptoms and burnout among Chinese medical staff of intensive care unit: The moderating effect of social support. BMC Psychiatry.

[43] Lai J., Ma S., Wang Y., Cai Z., Hu J., Wei N., Wu J., Du H., Chen T., Li R. (2020). Factors associated with mental health outcomes among health care workers exposed to coronavirus disease 2019. JAMA Netw. Open.

[44] Rümeysa Y.E., Ayşe K., Selim A., Emrah K. (2020). Depression, anxiety, stress levels of physicians and associated factors in COVID-19 pandemics. Psychiatry Res..

[45] Babicki M., Bogudzinska B., Kowalski K., Mastalerz-Migas A. (2022). Depression, Anxiety and Quality of Life among Online Responders in Poland: A Crosssectional Study Covering Four Waves of the COVID-19 Pandemic. Int. J. Environ. Res. Public Health.

[46] Saragih I.D., Tonapa S.I., Saragih I.S., Advani S., Batubara S.O., Suarilah S., Lin C.-J. (2021). Global prevalence of mental health problems among healthcare workers during the COVID-19 pandemic: A systematic review and metaanalysis. Int. J. Nurs. Stud..

[47] WHO Regional Office for Europe UN City, Marmorvej 51 DK-2100 Copenhagen OE, Denmark. Pandemic Fatigue: Reinvigorating the Public to Prevent COVID-19: Policy Framework for Supporting Pandemic Prevention and Management: Revised Version November 2020. https://apps.who.int/iris/handle/10665/337574.

[48] Marcotrigiano V., Pattavina F., Blangiardi L., Salerno G., Dalena A., Del Bianco F., Di Fant M., Fabbro A., Forgiarini M., Lanzilotti C. (2022). The Preventive Health Professions in Italy: The Efficient Use of Resources, Skills and Best Practice during the Pandemic. Healthcare.

